# Mothers’ perceptions of their own diets and the diets of their children at 2–3 years of age

**DOI:** 10.1186/s41155-017-0067-7

**Published:** 2017-06-12

**Authors:** Mônica Cristina Broilo, Márcia Regina Vitolo, Lucia Marques Stenzel, Daniela Centenaro Levandowski

**Affiliations:** 1 Graduate Program in Health Sciences, Federal University of Health Sciences of Porto Alegre—UFCSPA, Sarmento Leite 245, Porto Alegre, RS 90050-170 Brazil; 2 Department of Nutrition, Federal University of Health Sciences of Porto Alegre—UFCSPA, Sarmento Leite 245, Porto Alegre, RS 90050-170 Brazil; 3 Department of Psychology, Federal University of Health Sciences of Porto Alegre—UFCSPA, Sarmento Leite 245, Porto Alegre, RS 90050-170 Brazil

**Keywords:** Mothers, Perception, Child, Women, Food habits

## Abstract

This is a cross-sectional analysis of a follow-up study to examine the perceptions of mothers treated at public health centers, regarding their own diets and the diets of their children aged 2–3. Among the 464 participants, 57% (*n* = 267) reported perceiving their own diets as unhealthy while 72% (*n* = 334) perceiving their children’s diets as healthy. The mothers’ perceptions of their own diets as healthy were associated with less maternal schooling and having received health care from professionals who had received special training (*p* < 0.05). The mothers’ perceptions of their children’s diets as healthy were associated with more maternal schooling (*p* < 0.05). This difference between the mothers’ perceptions of their own diets and those of their children reinforce the importance of considering maternal beliefs and attitudes in infant nutritional intervention programs.

## Background

Diet plays an important role in the health of individuals from childhood to adulthood, as it influences factors such as growth, development, and metabolic programming. It also has an impact on the development of certain diseases in childhood and even in adulthood (Michaelsen, Larnkjaer, Lauritzen, & Mølgaard, 2010). Thus, it becomes relevant to study children’s dietary practices starting from birth, since these habits are formed in the first years of life and change little over time (Skinner, Carruth, Wendy, & Ziegler, 2002).

Regarding the process of establishment of eating habits, although children have innate preferences for certain tastes (Birch, 1998; Schwartz, Issanchou, & Nicklaus, 2009), repeated exposure to specific foods can stimulate their acceptance, which can determine their eating habits (Forestell & Mennella, 2007; Mennella & Beauchamp, 2005). In addition, other behavioral factors may influence children’s food intake, especially the family food environment, feeding practices adopted in relation to breastfeeding, and weaning age (Blissett & Fogel, 2013). In later years, factors such as restricting certain foods, pressure to eat, and adopting strategies to encourage and reward are also seen as having an important influence on children’s eating habits (Blissett & Fogel, 2013; Kröller, Jahnke, & Warschburger, 2013; Jansen et al., 2012).

As is possible to see, family members serve as a model for children eating habits (Blissett & Fogel, 2013; Kröller et al., 2013; Rodgers et al., 2013; Jansen et al., 2012; Mennella & Trabulsi, 2012; Barroso, Roncancio, Hinojosa, & Reifsnider, 2012; Hart, Raynor, Jelalian, & Drotar, 2010). Family members are an important reference for the foods that are offered to children too. In fact, although children can decide whether to accept a certain food and how much to eat, it is generally the caretakers who determine which foods are offered to them (Mennella & Trabulsi, 2012). In this sense, parents can have both a positive and a negative influence on their children’s eating habits and dietary quality (Adamo & Brett, 2014).

In particular, studies have highlighted the important influence mothers have on their children’s diets in relation to various factors, such as food intake, as mothers’ diets are similar to those of their children in terms of the types of food consumed (Hart et al., 2010). Parental influence has also been identified on children’s eating behaviors, as the attitudes of mothers regarding their own diets as well as those of their children can determine their children’s behavior in relation to food (Kröller et al., 2013). Mothers’ nutritional status has also been associated with that of their children (Barroso et al., 2012). Similarly, mothers’ satisfaction with their bodies is reflected in their children’s satisfaction with their own bodies (Rodgers et al., 2013).

Given the importance of parental behaviors (especially those of mothers) on children’s eating habits, it is important to understand this phenomenon not only by investigating family dietary practices, which have been studied extensively in recent years (Blissett & Fogel, 2013; Hart et al., 2010; Kröller et al., 2013), but also by focusing on parental perceptions and beliefs that support and influence these practices (behaviors and choices). In psychology, perception is more than just the reception of a stimulus. It is also comprised of the attribution of meaning to such stimuli, and its importance lies mainly in the fact that people’s behavior is based on their interpretation of reality and not necessarily of reality itself (Michner, Delamater, & Myers, 2005).

It is known that perception influences decision making in different areas such as health behaviors, including behaviors related to eating (Wang, Worsley, & Cunningham, 2008; Giles, McClenahan, Cairns, & Mallet, 2004; Stafleu, Van Staveren, De Graaf, Burema, & Hautvast, 1995). Besides, the literature shows that behavioral changes occur when an individual believes that her behavior is inappropriate and perceives that maintaining such behavior may lead to health risks (Straub, 2014). This has been observed in relation to several health conditions, such as diabetes and hypertension (Wisting et al., 2016; Luder, Frede, Kirby, King, & Heaton, 2015; Sansbury, Dasgupta, Guthrie, & Ward, 2014). However, studies that focus on the perception of health behaviors in non-clinical contexts (as this study does) are still rare. Furthermore, studies associating patients’ socio-economic and/or demographic variables with their perceptions about health were not found.

The present study aimed to analyze mothers’ perceptions about their own diets and those of their children (aged 2–3) considering the criterion of being healthy or not, as well as to investigate potential associations between these perceptions and maternal/family characteristics. This study’s importance lies in the fact that it does not search what mothers do in relation to their own diets and those of their children (which has been a frequent subject of mother-child nutrition studies) but rather what mothers say and think about this topic (i.e., how they perceive their own behavior in this regard). Studying how mothers perceive their own diets and those of their children can help to understand the quality of children’s diets and their resulting health outcomes. Thus, the knowledge of this topic can improve the effectiveness of nutritional interventions, which is of prime importance in early childhood.

## Methods

### Design

It is a cross-sectional analysis of data collected in a follow-up study (Vitolo, Louzada, & Rauber, 2014). This major study aimed to assess the impact of an educational intervention conducted with public health center (PHC) professionals on health outcomes of children whose mothers were treated by these professionals during pregnancy (Vitolo et al., 2014).

### Participants, procedures, and instruments

The participants of the major study were selected during pregnancy at 20 PHC in Porto Alegre, Rio Grande do Sul, Brazil, in order to cover both the territorial organization established by the city and the sample size (*n* = 715 mother-baby dyads) that had previously been planned for the main project. These women were allocated in two groups: (1) the intervention group, which was comprised of pregnant women treated by professionals who received a 1-h training at their workplaces (PHC) on the “Ten steps for healthy feeding for Brazilian children from birth to 2 years of age” government program (Brazil, Ministry of Health, 2002), and (2) the control group, which was comprised of pregnant women who were treated by professionals who did not receive this training at PHC.

This government program is a joint initiative of Brazilian Health Ministry and Health Promotion and Protection Program of the Pan-American Health Organization (PAHO-WHO), which aims to enable health professionals to promote healthy feeding practices for children under the age of 2. It is important to point out that these pregnant women did not receive any type of direct intervention by the research team. Their participation in the larger study was meant to identify potential health outcomes for their children related to the assistance received during pregnancy by PHC professionals who have been trained or not. Among the present study’s final sample, 51.9% (*n* = 241) of the women were originally treated by health professionals who received the training.

The data collection was done by a previously trained team composed of undergraduate and post-graduate students in nutrition and psychology. They identified women at the PHC who were in their third trimester of pregnancy.

In the first contact, the women were informed of the study’s procedures and objectives and were invited to participate, signing informed consent forms. They also responded to a questionnaire specifically prepared for the present study, in which they provided socio-economic, demographic, and family data. Information was also collected on expected birth date, address, and telephone number for subsequent home visits. HIV-positive women were excluded from the study. In the following data collection phases (when the children were aged 6–9 months, 12–16 months, and 2–3 years), the mothers were re-contacted and invited to participate again. The data collection for these three phases was conducted at the participants’ places of residence. A specific questionnaire was prepared for each data collection phase according to the child’s age group. At these times, the interviewees generally responded to questionnaires specifically prepared for use in the project that contained data about the child’s birth and state of health as well as his/her anthropometric and dietary data.

For the present study, we considered the data collected in the major project’s fourth collection stage (with mothers of children aged 2–3) regarding the mothers’ perceptions of their own diets and those of their children, which were specifically collected at this time. More specifically, the following questions were used to analyze maternal perceptions: (1) “Do you believe that your diet is healthy? If so, why? If not, why not?” and (2) “Do you believe that your child’s diet is healthy? If so, why? If not, why not?” The mothers were not specifically asked about their diets or the diets of their children (such as the types and quantities of foods consumed) or about what they believed to be a healthy diet. They responded to these two questions freely and only mentioned foods or concepts about a healthy diet when they wanted to.

The mothers’ answers regarding these questions were registered verbatim by the data collection staff. Family socio-economic and demographic data collected during pregnancy (a period corresponding to the main study’s first data collection phase) were also considered.

### Data analysis

All data collected were entered into a database and subjected to double entry in the Statistical Package for Social Science v.19.0 software program. The data were validated with the Epi Info v.6.4 program. The closed questions “Do you believe that your diet is healthy?” and “Do you believe that your child’s diet is healthy?” were quantified and presented in absolute frequencies. Answers to the open questions “If so, why?” and “If not, why not?” were classified using content analysis (Bardin, 2004). The responses were first transcribed verbatim. A pre-analysis was done with an initial reading of the participants’ answers and the formulation of preliminary categories according to their content. In the next stage, the material was explored and the responses were read again and classified into the initially created categories. During this process, the categories were reviewed by three judges to assess their suitability and mutual thematic exclusion. Whenever the categorical structure was found to be satisfactory, a judge would again assign the responses to their respective categories. Questions about this new classification were settled by discussion and consensus with the other two judges. Finally, the response categories were quantified in relative frequencies.

Any associations between personal/family characteristics and the mothers’ perceptions of their own diets and those of their children were verified using univariate Poisson regression with robust estimation. Variables with *p* ≤ 0.20 were analyzed using multivariate Poisson regression with robust estimation to independently verify their association with the mother’s perceptions. The significance level was considered to be 5%.

## Results

We interviewed 474 women for the present study. Of these, 10 were excluded because the questionnaire was not answered by the mother directly, but rather by >another caretaker responsible for the child (usually the maternal grandmother). Thus, the sample was comprised of 464 mothers. Of these mothers, 82.3% (*n* = 382) were aged 20 years or older when their babies were born, 65.5% (*n* = 304) did not have formal employment, 53.4% (*n* = 248) had 8 years of schooling or more, 77.2% (*n* = 435) lived with a husband or partner, 53.5% (*n* = 244) had a total family income higher than two minimum wages (Brazilian minimum wage corresponds an amount of U$130.00, approximately for month at the time of data collection), and 56.5% (*n* = 262) already had other children.

Among the maternal and family characteristics, the only one that differs significantly (*p* < 0.05)—comparing the present study’s sample size (fourth data collection stage) with the larger study’s initial sample (*n* = 715 mother-baby dyads)—is maternal age, due to the fact that there was a greater prevalence of follow-up loss among participating mothers under the age of 20. The other characteristics were significantly similar, demonstrating that the present study’s sample was still highly representative (*p* > 0.05).

Among the 464 women interviewed for this study, when evaluating their own diets (Table 1), 57% (*n* = 267) of the mothers reported that they did not consider them to be healthy. Factors related to a lack of diet quality were the most cited to account for this perception. A lack of regularity or routine, insufficient or excessive food intake, perceived health outcomes, and limited food variety were cited in different proportions to justify this perception, as shown in Table 1.

**Table 1:** Categorization and distribution of responses regarding mothers’ perceptions of their own diets (*n* = 464)

Perception of own diet as healthy (*n* = 197, 43%)			Perception of own diet as unhealthy (*n* = 267, 57%)		
Themes/examples of responses	*n*	%	Themes/examples of responses	*n*	%
*Quality* “Because I don’t drink sodas or eat junk food” “I avoid unhealthy foods, I avoid oils” “I eat healthy things” “Because I try to eat little sugar and salt” “I don’t eat much fried food and I don’t mix carbohydrates” “I eat homemade food”	135	70.7	*Quality* “I eat a lot of bread” “I don’t like fruits and vegetables much” “I eat a lot of junk food” “Because I eat more junk food than healthy food”	186	74.1
*Variety* “I eat everything” “I eat everything, I’m not picky” “Because I try to vary my diet”	28	14.7	*Regularity or routine* “I don’t eat lunch or breakfast” “I don’t have a fixed eating schedule, I don’t eat breakfast” “I eat few meals during the day”	75	29.9
*Regularity or routine* “I am eating regularly” “I am eating all the time” “I eat breakfast, lunch and dinner” “I make lunch and dinner every day”	17	8.9	*Quantity* “I eat very little” “I eat way too much” “I don’t eat much…”	20	08
*Perceived health outcomes* “Because I go to the doctor and I don’t have any problems” “I’m trying to lose weight”	15	7.9	*Perceived health outcomes* “After finding out I have cancer, I haven’t eaten properly” “I’m about 10 kg overweight”	09	3.6
*Does not know* “I don’t know” “It’s healthy but I don’t know why”	11	5.8	*Variety* “Because I spend the day eating bread and coffee” “Because I’m not used to it and I don’t like some foods” “I only eat the basics: rice and beans”	03	1.2
*Quantity* “I eat normally, I usually don’t eat much” “Because I eat a lot” “I don’t eat much”	07	3.7	*Does not know* “I don’t know”	02	0.8

Data lacking (*n* = 22): mothers who did not answer the question: “Do you consider your diet to be healthy? If so, why? If not, why not?”

On the other hand, 43% (*n* = 197) of the mothers considered their diets to be healthy. Among the reasons for this perception, those related to the quality of food consumed were the most cited by the respondents. In addition, these mothers believed that factors such as the variety of foods consumed, the regularity or routine of consumption, perceived health outcomes, and the amount of food consumed also determined their perceptions of their own diets as being healthy (Table 1).

When the mothers talked about their perceptions of their children’s diets, a different overview was observed, as 72% (*n* = 334) considered them to be healthy. However, as with their own diets, factors related to the quality of the foods consumed were most cited by the participants to determine this perception. The mothers cited variety, regularity/routine, school meals, perceived health outcomes, and amount of food consumed (Table 2) as reasons for this positive perception. Among the respondents who did not consider their children’s diets to be healthy (28%, *n* = 130), a lack/deficiency of diet quality was most frequently reported, while amount of food, regularity/routine, variety of foods consumed, and perceived health outcomes were cited less frequently (Table 2).

**Table 2:** Categorization and distribution of responses regarding mothers’ perceptions of their children’s diets (*n* = 464)

Perception of child’s diet as healthy (*n* = 334, 72%)			Perception of child’s diet as unhealthy (*n* = 130, 28%)		
Themes/examples of responses	*n*	%	Themes/examples of responses	*n*	%
*Quality* “He always eats vegetables and meat” “He avoids salty snacks, ‘snacks, soft drinks, instant noodles” “Because he eats the main foods with iron and vitamins” “Because I try to give him healthy food”	210	68.6	*Quality* “He eats a lot of fried foods” “He should have more fruits and vegetables” “Because he eats a lot of junk food” “He doesn’t eat well, he was eating a lot of snacks, sweets, junk food”	95	78.5
*Variety* “He eats everything” “He eats everything, he’s balanced” “He eats everything, he doesn’t refuse anything”	46	15	*Quantity* “Because he doesn’t eat anything” “He only eats a little of everything” “Because he doesn’t eat enough to be healthy”	14	11.6
*Regularity or routine* “He eats breakfast, lunch, an afternoon snack and, before going to sleep, drinks from his milk bottle” “Because he eats at the time” “Because he eats and makes all the meals of the day”	26	8.5	*Variety* “He doesn’t eat everything he should” “Because he doesn’t eat anything, practically only milk” “He only likes to drink milk”	12	9.9
*School meals* “Because he eats well at school” “Meals aren’t made at home, only at school”	21	6.9	*Regularity or routine* “Because there is no routine for the preparations, for making vegetables every day” “He doesn’t have a regular diet”	12	9.9
*Perceived health outcomes* “Because he is gaining weight” “Because he is always well when he sees the doctor”	17	5.6	*Perceived health outcomes* “He has a stomach ache” “He is lactose-intolerant”	03	2.5
*Quantity* “He eats a lot” “Because he eats well, he eats a lot” “Because he eats everything”	16	5.2	*Does not know* “I don’t know”	02	1.7
*Does not know* “I don’t know”	06	02			

Data lacking (*n* = 37): mothers who did not answer the question: “Do you consider your child’s diet to be healthy? If so, why? If not, why not?”

Personal and family characteristics were subsequently associated with the mothers’ perceptions about their own diets (Table 3). The perception of having a healthy diet was significantly associated with originally belonging to the group that was treated by trained PHC professionals during pregnancy and having less than 8 years of schooling (*p* < 0.05). On the other side, the mothers’ perception that their children’s diets were healthy was associated with having 8 or more years of schooling (*p* < 0.05), as shown in Table 4.

**Table 3:** Association between mothers’ perception of their own diets and personal and family characteristics (*n* = 464)

	Mothers’ perception of diet as healthy (*n* = 197, 43%)		Univariate Poisson regression			Multivariate Poisson regression		
	*n*	%	PR	95% CI	*p* value	PR	95% CI	*p* value
Group^a^								
Control	82	36.8						
Intervention	115	48.9	1.331	1.072–1.652	0.010	1.320	1.063–1.640	0.012
Age								
<20 years	35	43.2						
≥20 years	162	43	0.994	0.755–1.310	0.969	–	–	–
Schooling								
<8 years	107	49.8						
≥8 years	90	37	0.744	0.602–0.920	0.006	0.707	0.569–0.880	0.002
Marital status								
Does not have partner	51	48.6						
Has partner	146	41.4	0.852	0.675–1.075	0.176	0.918	0.723–1.164	0.479
Mother’s occupation								
No formal employment	121	40.5						
Formal employment	76	47.8	1.181	0.955–1.461	0.125	1.225	0.987–1.519	0.065
Family income^b^								
<2 Brazilian minimum wages^c^	88	42.9						
≥2 Brazilian minimum wages^c^	101	42.4	0.989	0.796–1.227	0.917	–	–	–
Parity								
Primiparous	83	41.9						
Multiparous	114	43.8	1.046	0.844–1.296	0.681	–	–	–

*PR* prevalence ratio, *CI* confidence interval

^a^Group in which the mother-child pair was assigned to the follow-up study (to receive guidance from updated professionals or control group professionals)

^b^Data lacking (*n* = 8)

^c^1 Brazilian minimum wage corresponds an amount of U$130.00 for month at the time of data collection

**Table 4:** Association between mothers’ perception of their children’s diets and personal and family characteristics (*n* = 464)

	Maternal perception of child’s diet as healthy (n = 334, 72%)		Univariate Poisson regression			Multivariate Poisson regression		
	*n*	%	RP	95% CI	*p* value	RP	95% CI	*p* value
Group^a^								
Control	154	69.1						
Intervention	180	74.7	1.082	0.964–1.213	0.180	1.063	0.946–1.194	0.306
Age								
<20 years	52	63.4						
≥20 years	282	73.8	1.164	0.977–1.387	0.089	1.139	0.951–1.364	0.158
Schooling								
<8 years	143	66.2						
≥8 years	191	77	1.163	1.035–1.308	0.011	1.136	1.011–1.278	0.033
Marital status								
Does not have partner	80	75.5						
Has partner	254	70.9	0.940	0.828–1.068	0.341	–	–	–
Mother’s occupation								
No formal employment	213	70.1						
Formal employment	121	75.6	1.079	0.962–1.210	0.192	1.035	0.919–1.165	0.575
Family income^b^								
<2 Brazilian minimum wages^c^	150	71.8						
≥2 Brazilian minimum wages^c^	176	73.3	1.022	0.911–1.145	0.712	–	–	–
Parity								
Primiparous	151	74.8						
Multiparous	183	69.8	0.934	0.835–1.046	0.239	–	–	–

*PR* prevalence ratio, *CI* confidence interval

^a^Group in which the mother-child pair was assigned to the follow-up study (to receive guidance from updated professionals or control group professionals).

^b^Data lacking (*n* = 8)

^c^1 Brazilian minimum wage corresponds an amount of U$130.00 for month at the time of data collection

## Discussion

This study aimed to analyze mothers’ perceptions of their own diets and those of their children aged 2–3 years and also to investigate associations between these perceptions and maternal/family characteristics. The results indicate differences between mothers’ perceptions of their own diets and those of their children as being healthy or not, although the reasons cited by the participants to explain such perceptions were quite similar, especially in reference to diet quality (i.e., the types of foods consumed). In order to better explore the data, this section was divided in two parts.

### Maternal perceptions of their own diets

Regarding this topic, most mothers (57%) did not consider their diets to be healthy, which is similar with results of a study conducted with a representative Brazilian sample (Instituto Brasileiro de Geografia e Estatística [IBGE], 2010). According to this national survey, the diets of adult women are not compatible with healthy eating, because fruit, vegetable, and fiber intakes are generally insufficient and various vitamin and mineral intakes are below recommended levels. In addition, a high consumption of saturated fat, sodium, sugar, and sugary drinks was observed (Instituto Brasileiro de Geografia e Estatística [IBGE], 2010).

In this study, although women were encouraged to express themselves freely about the reasons for why they considered or not their own diets as healthy, it is possible to note in their answers the parameters usually employed to classify dietary adequacy, such as quality (types of foods), quantity, variety, and regularity (Kennedy, Ohls, Carlson, & Fleming, 1995). One reason for the participants’ proper identification of these aspects related to healthy eating may be the fact that this information has been widely reported by mass media and, in this way, has become common sense for most people. In fact, the interviewees demonstrated a minimally correct knowledge of the parameters employed to evaluate diet quality.

Analyzing the interviewees’ responses, it was possible to note that diet quality (in terms of the types of food consumed) was the most representative factor in evaluating their own diets as being healthy or not. The same mothers attributed their compromised dietary quality to the consumption of foods of low nutritional value. In addition, the interviewees highlighted the importance of the types of food eaten. Fruits, vegetables, rice, beans, and meat were considered to be beneficial and responsible for a healthy diet, as opposed to fried foods, snacks, sweets, treats, or even a lack of the previously cited “healthy” foods. The great value attributed to natural food over processed and ultra-processed ones is in agreement with proposals from the Food Guide for the Brazilian Population, which incorporates values from the country’s traditional food culture, highlighting the importance of a diet based on fresh natural food (Brazil, 2014).

Therefore, the interviewees’ perceptions about what types of food are healthy proved to be appropriate, according to scientifically recognized dietary patterns (Brazil, Ministry of Health, Secretary of Health Care, Department of Primary Care, 2014). However, as there are multiple aspects involved in eating behavior, perhaps the interviewees cannot classify their diet quality in a fully accurate manner, possibly due to a lack of information or even critical thinking about the subject, or because of other social and cultural aspects that are intertwined in these choices, but were not investigated here. These aspects may have contributed to the fact that the mothers were not able to effectively implement a healthy diet.

In fact, to know the parameters is not a guarantee of healthy eating habits. Even though mothers may know that factors such as quality, quantity, and variety are important to a healthy diet, it is possible to think that they are not always able to identify what kind of food are really healthy, what are the appropriate quantities recommended for themselves or their children, and how varied a diet should be to be effectively considered healthy. In this direction, a qualitative study conducted with mothers of school children, with a socio-economic level similar to those of the present study, identified that, although the mothers recognized the importance of healthy lifestyle habits and a healthy diet for their own health and that of their children, they had considerable difficulty in identifying what was contemplated within this concept, regardless of their educational level (Oli, Vaidya, Subedi, Eiben, & Krettek, 2015). In that study, they showed to have some appropriate concepts, such as considering ultra-processed foods to be incompatible with a healthy diet, also observed in the present study, as well as others that were inappropriate or contradictory, such as considering a healthy diet to be bland or appropriate only for sick people (Oli et al., 2015).

These difficulties could explain the fact that, although 43% of mothers mentioned having a healthy diet, it was possible to identify perceptions not fully consistent with healthy eating patterns based on some of the mothers’ statements (see Table 1). The literature has showed that many factors influence people’s food choices, including aspects related to food itself, such as its nutritional characteristics, price, flavor, variety, and availability, as well as factors related to the individual such as biological, socio-cultural, anthropological, economic, and psychological determinants (Jomori, Proença, & Calvo, 2008). Therefore, one might think that the mothers interviewed actually considered health-related issues when they chose foods and consequently reported that they believed their diets to be healthy. However, even though they judged the foods they chose to be suitable for a healthy diet, many other factors (including those mentioned above) act simultaneously, determining the real mothers’ food choices, possibly without them having considered this issue, what could explain some patterns that are not fully consistent with a healthy diet identified in these women’s daily practices.

The discrepancy found between the mothers’ assessment of their diets and some of the statements they made can also lead one to assume that they responded to the questions based on what they believed to be socially expected and therefore socially desirable behavior. Health Psychology Theories (Fishbein & Ajzen, 1975; Ajzen & Madden, 1986) point out that part of the attitude related to behavior is based on a subjective norm, which consists of the interpretation an individual makes about being approved of or not by another person in regard to a certain behavior. Perhaps the mothers thought that the interviewers would appreciate more responses that indicated healthy diets for themselves and especially for their children, who were the study’s primary focus.

Despite the hypothesis mentioned above, with respect to the association analyses, the mothers’ perceptions of their own diets being healthy were significantly more prevalent among the participants who originally belonged to the group that received care from trained PHC professionals during pregnancy. A previous study, which was conducted with the same sample when their children were aged 6–9 months, found that the mothers who reported following the health professionals’ guidelines adopted much healthier feeding practices for their children (Broilo, Louzada, Stenzel, & Vitolo, 2013). This data may be related to the association found in the present study, in that the women who were counseled by trained professionals and who were consequently much better prepared probably adopted healthier feeding practices for their children and possibly for themselves. This finding indicates not only that parents influence their children’s eating habits but also that children can influence the food choices that mothers make for themselves (Guidetti & Cavazza, 2008), because the guidelines of these professionals were directed to infant eating practices. To some extent, this finding is consistent with the previously cited social desirability hypothesis, as these mothers may have more strongly perceived the professionals’ emphasis on taking care of child’s diet and, in consequence, on their own diets.

Formal education was another maternal characteristic significantly associated with the perception the mothers had of their own diets being healthy. Particularly, women with less than 8 years of schooling reported having this perception more frequently. This result could be explained by the fact that these mothers can be less informed and less critical about the criteria that would define a diet as healthy. In fact, there are many factors that can influence an individual’s food choices for herself and for her family (Adamo & Brett, 2014) and a higher level of education can ensure a better diet, but not necessarily a positive perception of it. On the contrary, it leads to being more critical of one’s eating habits. So, less years of formal education could interfere on this evaluation due to restrict knowledge and less critical thinking about this topic.

### Maternal perceptions of their child’s diets

Contrary of the results about their own diets, when asked about their perceptions of their children’s diets, 72% of the participants considered them to be healthy. A similar result was found in a study that compared mothers’ perceptions of their children’s diets with the children’s actual diet quality. Although only 0.2% of the children had diets considered good, 78% of the mothers reported believing that their children’s diets were adequate (Kourlaba, Kondaki, Grammatikaki, Roma-Giannikou, & Manios, 2009). Although the present study did not assess the food actually consumed by the children (since it focused on mothers’ perceptions), national review studies (Carvalho, Fonsêca, Priore, Franceschini, & Novaes, 2015) using similar samples (Sparrenberger, Friedrich, Schiffner, Schuch, & Wagner, 2015), and other studies that considered data from the sample’s main project from which the present study is derived (Valmórbida & Vitolo, 2014), indicate that children’s diets fall well short of recommendations and do so at increasingly younger ages. Thus, it is noted that mothers’ perceptions of the healthy feeding of their children may not be consistent with the food their children actually receive, as is indicated in the literature (Kourlaba et al., 2009).

This finding bears highlighting because, in order to change a behavior, it is necessary for an individual to recognize that a diet is not adequate and/or perceive the health risks of maintaining such a diet (Straub, 2014). This fact was demonstrated in a review study that highlighted a mother’s perception of her child as being obese as a necessary requirement for her to seek professional help and to follow nutritional guidelines (Camargo, Barros Filho, Antonio, & Giglio, 2013). In this regard, the present study’s findings enable a partial understanding of the reasons for rising levels of child nutrition inadequacy found in a Brazilian sample, especially when related to overweight and obesity (Instituto Brasileiro de Geografia e Estatística [IBGE], 2010). Our results confirms the low probability of changing dietary behaviors or even seeking professional assistance to discuss child feeding issues, because a consideration of being healthy.

With regard to the reasons reported by the mothers to justify their perceptions of their children’s diets as healthy or not, the same categories as those previously cited were identified. Diet quality (with an emphasis on the types of foods consumed) was again used as the criterion that guided such perceptions. It was possible to observe similarities in the values the mothers attributed to some specific types of food, both in relation to their children’s diets and to their own.

In the case of the mothers who consider their children’s diets to be healthy, it is worth noting their emphasis on the important role of natural and traditional foods in Brazilian cuisine, such as rice, beans, meat, fruits, and vegetables. These mothers reported that they considered their children’s diets to be healthy because they consumed these types of foods. Among the mothers who did not consider their children’s diets to be healthy, ultra-processed foods and foods of low nutritional value such as salty snacks, sweets, soft drinks, and sugary artificial juices were presented as being responsible for the compromised diet quality. Thus, it can be assumed that healthy feeding was analyzed by most of the mothers in a one-dimensional manner, observing only one of a diet’s characteristics—in this case, its quality (the types of foods provided). This finding agrees with the potential difficulty (mentioned above) of considering all of the aspects involved in the concept of healthy eating, as is indicated in the literature (Oli et al., 2015). The mothers appear to select a specific criterion and not to consider others that are potentially relevant.

Another finding that merits attention is the large difference observed in our study between the mothers’ perceptions of their own diets and those of their children. This could be explained by the fact that the mothers may not perceive inadequacies in their children’s diets as clearly as they do with respect to their own dietary behavior. In this sense, a comparison could be made with the difficulty mothers have in identifying problems related to their children’s nutritional state. National and international studies have shown that, although women can identify their own nutritional status properly, they have great difficulty in identifying their children’s nutritional status adequately, especially in cases of overweight or obesity (Francescatto, Santos, Coutinho, & Costa, 2014; Guerrero, Slusser, Barreto, Rosales, & Kuo, 2010; Boa-Sorte et al., 2007; Hackie & Bowles, 2007).

It is possible that the mothers had difficulties perceiving problems with the food provided to and/or consumed by their children, as affirming that their children’s diets are not healthy can be seen as recognition by these mothers that they are providing low-quality food to their children. This reflection brings up historical and social questions about the role of the mother, who has been assigned the responsibility of providing nutrition to her child. Women have been held responsible and blamed (even by themselves) when something happens that is not socially desirable or expected (Moura & Araújo, 2004; Ramos & Almeida, 2003). Thus, it is possible to believe that affirming that a child’s diet is not healthy would be equivalent to admitting (explicitly or not) to an inappropriate maternal behavior worthy of reprimand or blame. This is especially true in regard to diet, which is associated with a general state of health and imbued with affective connotations (Rotenberg & Vargas, 2004).

Finally, the significant association found regarding the mothers’ perception that their children’s diets are healthy with maternal schooling of 8 years or more may suggest that mothers with more schooling believe that their children’s diets are healthier because they possibly have more information and greater discernment about the subject and are therefore able to analyze other aspects related to diet quality besides merely the types of food their children eat. The present study did not analyze the quality of the children’s diets. However, studies conducted in Brazil (Saldiva et al., 2014) and other countries (Brekke, Van Odijk, & Ludvigsson, 2007) have demonstrated an association between a higher maternal educational level and a higher diet quality for their children in regard to several aspects, such as greater fruit and vegetable consumption and reduced consumption of food with low nutritional value. It is worth emphasizing that the fact that mothers with more schooling more commonly report that their children’s diets are healthy does not necessarily correspond to the food provided to their children, as we commented about mothers diets. However, one can assume that these mothers have more physical and financial resources to provide their children with appropriate food.

The fact that the mothers with more schooling more frequently reported the perception that their children’s diet was healthy while the association was inverse when reporting on their own diets (i.e., they more frequently reported the perception that their own diet was not healthy) may initially seem discrepant but plausible. Assuming that mothers with more schooling have greater knowledge and discernment about the subject, this enables them to recognize shortcomings in their diet (more frequently than mothers with less schooling) and this recognition can help to increase the quality of the foods offered to their children, which would explain the fact that they more often evaluated their children’s diet as healthy.

In analyzing the present study’s qualitative data, it is possible to find testimonials that corroborate this hypothesis, in which mothers with more schooling are critical of their diets, recognizing that their diets have shortcomings. However, at the same time, they offer healthy foods to their children: “I don’t usually eat lunch or dinner. I only want to eat breakfast. I don’t like fruits or salads very much.” (her own diet) and “I make her eat everything. Even if she doesn’t like it, I give it to her.” (her child’s diet). Another interviewee, when talking about her diet, said “I apply something to my children that I am not able to follow. I like fast food.” In regard to her children’s diet, she said “She eats quite varied meals. She eats lots of vegetables and fruits.”

## Conclusions

This study contributes to a better understanding of women’s perceptions of their own diets and the diets of their 2 to 3-year-old children, considering the criterion of being healthy or not. The differences observed among these perceptions indicate a need for a differentiated approach in caring for the nutrition of both mothers and children. It is important to clarify that, although the nutritional needs of each age group are different, a healthy diet can be generalized for all family members. Furthermore, it is necessary to approach each person individually, considering his/her perceptions and beliefs, in order to provide effective and individualized care.

The differences found in the present study between the mothers’ perception of themselves and of their children in relation to the quality of their diets and the factors associated with these differences can lead to a reflection on the different female roles as woman and mother (Moura & Araújo, 2004) and about how the interviewees may think and behave differently about their own diets and the food they provide to their children, as well as about other health and general care behaviors, regardless of socio-economic or demographic characteristics.

Therefore, it is important to conduct more studies on this topic to investigate the subjective meanings of mothers’ attributions to their children’s diets. This type of study could help professionals identify the mechanisms involved in the process of recognizing behaviors related to children’s diets and health. The importance of such a detailed investigation lies in the fact that the failure to recognize children’s eating behaviors leads to a predisposition to diet-related diseases caused by excessive amounts of food and/or a lack of nutrients in a child’s diet (Adamo & Brett, 2014).

One of the present study’s potential limitations is the fact that it did not analyze the relationships between maternal perceptions of their own diets and those of their children and the dietary and/or nutritional data of the sample in question. However, this was a methodological choice, as the dietary characteristics of Brazilian children in this age group have already been frequently analyzed (Carvalho et al., 2015; Valmórbida & Vitolo, 2014), as opposed to maternal perceptions about the subject which, as the present study shows, should be considered by professionals who provide health care to children and their families.

Another limitation of the study was the fact that differences in mothers’ perceptions of infant healthy diets according to the child’s sex were not analyzed. Considering gender differences and its impacts on raising children, we think that it is an important topic for future studies, as well the analysis of possible contextual influences on the mothers’ perceptions about diets, such as social representations about health and beauty.

Therefore, it can be considered a strong point that this study focuses on a non-clinical group of mothers and on their perceptions of their own diets and those of their children in early childhood. The ability of parents to accurately identify what a healthy diet is can be essential to the success of programs to promote early healthy eating habits for this age group. This knowledge can assist health professionals in more effectively counseling people in the public health context as they deal much more with patients’ perceptions than with their actions. Understanding and intervening in regard to perceptions gives health professionals a greater chance to guide patients in modifying their dietary behaviors.

As was highlighted in a review study (Adamo & Brett, 2014), there are many parental perceptions related to children’s diets that may not be compatible with scientific criteria. Therefore, studies on this topic are still quite necessary (Adamo & Brett, 2014), and it could be done not only by quantitative studies. Qualitative design seem to be an important choice to understand maternal perceptions and practices and, at the end, the infant eating habits and nutritional outcomes. In fact, all efforts must be directed at improving children’s diets, mainly due to their impact on growth and development beyond childhood; therefore, we re-emphasize the importance of this type of investigation, since to invest in actions aimed at improving children’s health is to invest in the quality of life of the whole population.
